# Metastatic Renal Cell Carcinoma Presenting as Prolonged Pyrexia and Stauffer's Syndrome: Can a Routine Ultrasound Scan Fail to Detect a Renal Cell Carcinoma?

**DOI:** 10.1155/2018/4215041

**Published:** 2018-07-02

**Authors:** C. L. Fonseka, A. G. T. A. Kariyawasam, S. A. G. L. Singhapura, C. M. de Silva, T. E. Kanakkahewa, I. G. T. M. Senarathna, C. K. Bodinayake

**Affiliations:** ^1^University Medical Unit, Teaching Hospital Karapitiya, Galle, Sri Lanka; ^2^Department of Internal Medicine, Faculty of Medicine, University of Ruhuna, Sri Lanka

## Abstract

**Background:**

Prolonged pyrexia and weight loss are recognised paraneoplastic manifestations of renal cell carcinoma (RCC). Stauffer's syndrome is a rarely described paraneoplastic manifestation, which is described early in the course of RCC. We report a patient who presented with unresolving fever with multiple pulmonary opacities with biochemical evidence of hepatic choleastasis and was later diagnosed to have metastatic RCC with Stauffer's syndrome.

**Case Presentation:**

We report a 54-year-old female who was investigated for a poorly resolving fever and recent weight loss for two months. During her course of illness, she developed bilateral multiple opacifications in the chest radiograph with negative pyogenic, mycobacterial microbiological studies. Despite intravenous antibiotics, her fever continued. She was found to have elevated alkaline phosphatase and gamma-glutamyl transferase and she underwent imaging with ultrasound scan of abdomen twice, which did not reveal demonstrable abnormalities. Later, contrast CT of abdomen and chest was performed and detected a renal cell carcinoma of the right upper pole of the kidney with multiple lung metastases, which was concluded as a metastatic RCC with paraneoplastic Stauffer's syndrome.

**Conclusion:**

Prolonged pyrexia with loss of weight and Stauffer's syndrome could be features to suggest renal cell carcinoma in the absence of positive microbiological studies. Isoechoic RCC could be missed in routine ultrasonography. When a RCC is suspected in the setting of a pyrexia of unknown origin, ultrasound with doppler or a contrast CT should be requested to aid diagnosis.

## 1. Background

Renal cell carcinoma (RCC) accounts for 2% of cancers worldwide. The incidence of RCC has risen over the past several decades largely due to incidental detection by imaging modalities such as CT scan, ultrasound, and MRI [1], in which the accuracy of diagnosis with CT scan amounts to greater than 95% [2]. The most common clinical presentations of RCC are hematuria (50-60%), abdominal pain (40%), and a palpable abdominal mass (30%); this classic triad of symptoms is present in less than 10% of cases [3]. Paraneoplastic manifestations of RCC, including hypercalcemia, polycythemia, hepatic dysfunction, amyloidosis, fever, and weight loss, are present in up to 20% of patients [4].

A paraneoplastic syndrome may be the initial clinical presentation of RCC in a significant number of patients, and recognition of these syndromes may facilitate early diagnosis. Most paraneoplastic syndromes associated with RCC remit after resection of the primary RCC or treatment of metastatic sites [4]. Hepatic panel abnormalities in the absence of hepatic metastasis, a condition known as Stauffer's syndrome, have been described as a paraneoplastic phenomenon classically in early-stage renal cell carcinoma (RCC) [5]. But Stauffer's syndrome has been anecdotally described with other cancers such as prostate cancer [6], bladder cancer [7], and pancreatic cancer [8] as well as bronchial malignancy [9].

Typical Stauffer's syndrome presents elevations in the enzymes aspartate transaminase (AST) and alanine transaminase (ALT) and alkaline phosphatase (ALP) without jaundice. However, atypical presentations with a jaundice component have also been described [10, 11]. The pathogenesis of Stauffer's syndrome is still incompletely elucidated. Blay et al. found that interleukin-6 (IL-6) levels are elevated in patients with RCC as well as elevated alkaline-phosphatase, bilirubin, and gamma-glutamyl transferase (GGT), suggesting a probable IL-6 mediated process [12]. Biopsy from the liver in this syndrome typically shows cholestasis, sometimes with a mild inflammatory infiltrate. Surgical removal of the otherwise local cancer via nephrectomy leads to resolution of the hepatic abnormalities. In cases where there was a recurrence of cancer, this was heralded by the resurgence of the liver enzymes [13].

We report a patient with RCC who presented with prolonged pyrexia and multiple pulmonary opacities with hepatic cholestasis. RCC was missed in routine ultrasound scan but was later diagnosed by a contrast CT scan.

## 2. Case

A 54-year-old previously healthy female presented to the hospital with intermittent low-grade fever for two months with a mild dry cough. There was no associated pleuritic chest pain, shortness of breath, or hemoptysis. Accompanying anorexia and weight loss were pronounced. She gave a recent history of being investigated for right side loin pain, where she was managed as right renal calculus, which was evident with the ultrasound scan. But she did not have urinary symptoms or hematuria. Examination revealed a female who looked ill. She was averagely built but claimed that she has been overweight previously. She was afebrile and had mild pallor. There were no enlarged lymph glands. Respiratory system was clinically normal without pleural effusions or added sounds. She had regular pulse rate of 72 bpm and blood pressure of 120/80 mmHg. She had no hepatosplenomegaly or ballotable loin masses. She gave a history of being treated by several doctors with antibiotics for a possible infection.

**Figure 1:**
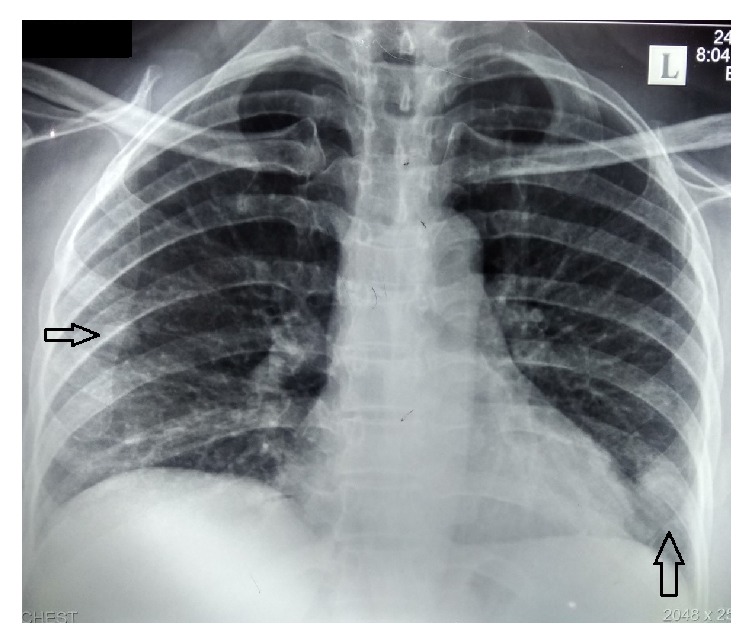
Bilateral multiple opacifications in chest radiograph.

Her complete blood count revealed normal white cell and platelet count. Her hemoglobin was 9.4 × 103/*μ*L with a normochromic normocytic anemia. Her inflammatory markers were significantly elevated with an ESR of 130 in the first hour and CRP of 124 u/l. Blood, urine, and sputum for pyogenic, mycobacterial, and fungal cultures were negative repeatedly, while the chest radiograph showed multiple bilateral opacities with small nodular lesions over all three zones of both lungs (Figure 1). Mantoux test revealed a wheal of 12 mm and the serology and cultures for melioidosis were negative. She had normal renal function tests with normal urine analysis. The liver functions revealed mildly elevated SGOT and SGPT (80/68 u/l) with markedly elevated ALP (417 U/L) and GGT (592 U/L). The total bilirubin was normal. Ultrasound scan of the abdomen was done twice and did not reveal a significant abnormality.

**Figure 2:**
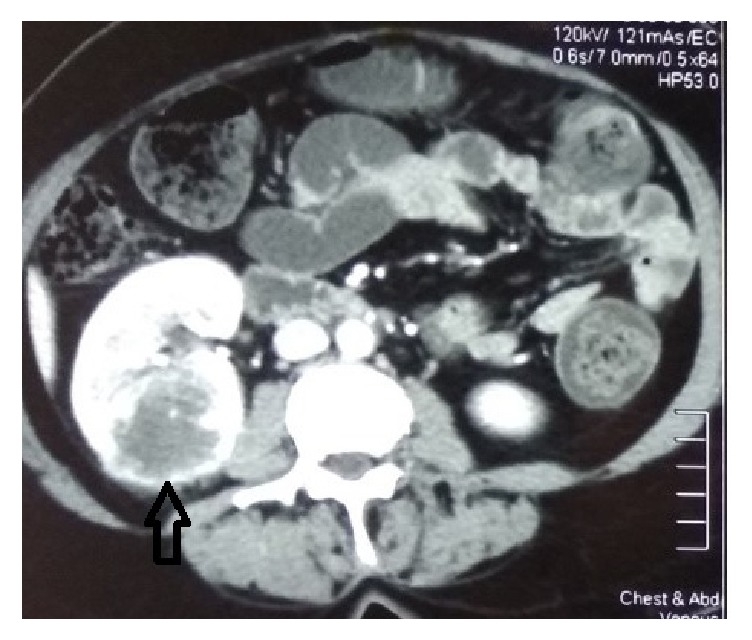
Renal cell carcinoma of the right kidney.

**Figure 3:**
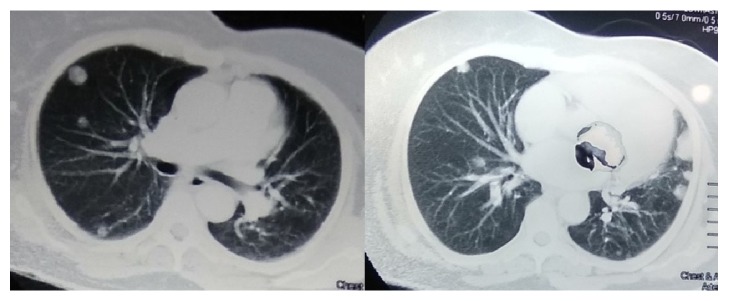
Bilateral pulmonary metastasis in middle and lower zones in CT of chest.

We empirically treated her for possible chest infection with intravenous antibiotics (ceftazidime) for two weeks. But neither the fever nor the inflammatory markers showed any response and the lung opacities were persistent. Next, we performed a contrast-enhanced computed tomography (CECT) of chest and abdomen. The CECT revealed a renal cell carcinoma of the right kidney (Figure 2) with multiple pulmonary metastasis (Figure 3).

She was transferred for oncology care and was treated with oral Sunitinib, a multitargeted receptor tyrosine kinase inhibitor, in the hope of subsequent nephrectomy. With treatment, the hepatic abnormalities resolved.

## 3. Discussion

Pyrexia and Stauffer's syndrome are paraneoplastic features that usually occur early in patients with RCC and are reported in several case reports [13, 14]. There is a report where Stauffer's syndrome has presented with metastatic renal cell carcinoma [14]. Our patient had persistent fever with multiple bilateral opacifications, which directed us to exclude subacute bacterial endocarditis, pulmonary tuberculosis, and pulmonary melioidosis. But the patient showed a poor response to intravenous ceftazidime and also had negative mycobacterial studies. Possibility of cholestatic hepatitis was excluded by doing hepatitis serology and she did not have any hepatic metastasis or bile duct dilatation in imaging. After detection of multiple opacities with a repeated negative ultrasound imaging, these directed us to perform a CECT of chest and abdomen in view of looking for lymphoreticular malignancy. This revealed a renal cell carcinoma in the right kidney with multiple pulmonary metastases.

Our patient had a RCC that was missed in routine US leading to a delay in diagnosis. Ultrasonography (US) in kidneys is usually useful in detecting solid from cystic masses with recent advanced US techniques. However, its use is still limited in detecting small lesion less than 10 mm and masses with similar echogenicity to the surrounding renal parenchyma. RCCs can have variable echogenic patterns that could be more echogenic, less echogenic, or isoechogenic. Isoechoic RCC may be difficult to detect with US, especially if it is small or displaces collecting system or gives rise to contour abnormalities. Also, isoechoic RCC can mimic normal anatomic structures or variants such as hypertrophy of ducts of Bertin [15]. Also, the tumour pseudocapsule can sometimes be visualised with ultrasound as a hypoechoic halo. Although this is a relatively specific sign, it is not particularly sensitive. Use of harmonic scanning has been reported to increase sensitivity to up to 85% [16]. Contrast-enhanced ultrasound may typically show heterogeneously hypervascular lesions in the arterial phase with early washout in the delayed phase [17]. Therefore, it should be emphasized that when there are isoechoic renal signals, US Doppler or other techniques in US could be used to detect increased and aberrant vascularity, which suggests the presence of a RCC. Also, calcifications in RCC could be mistaken for renal stones as in our case of an isoechoic RCC.

CT is optimal modality to detect RCC, and sometimes corticomedullary phases and nephrogenic phases are used to characterize the enhancement pattern of renal masses [15]. RCC can have variable densities in CT depending on areas of necrosis, hemorrhage, or calcifications. Rather than solid RCCs, cystic RCCs may show confusing radiographic patterns of malignancy and may be less specific than solid RCCs [18].

Therefore, radiographic imaging in RCC has certain pitfalls. The knowledge about these pitfalls is important to rectify these errors to arrive at an early diagnosis. Our patient had markedly elevated alkaline phosphatase and *γ*-glutamyl transferase with mild transaminitis. This case illustrates the importance of being familiar with Stauffer's syndrome in the background of prolonged fever, with a view to investigation for RCC.

## 4. Conclusion

Renal cell carcinoma can present as prolonged fever and Stauffer's syndrome even in an advanced stage of RCC with pulmonary metastasis. Ultrasonography can have certain pitfalls in detecting renal cell carcinoma. Isoechoic RCC could be missed in routine abdominal ultrasonography delaying diagnosis. CT scan would be a preferable choice in cases where obvious cause is unidentified in a setting of poorly resolving fever.

## Data Availability

All relevant data are included in the manuscript.
