# Corrigendum to “Metastatic Renal Cell Carcinoma Presenting as Prolonged Pyrexia and Stauffer's Syndrome: Can a Routine Ultrasound Scan Fail to Detect a Renal Cell Carcinoma?”

**DOI:** 10.1155/2018/6852737

**Published:** 2018-10-29

**Authors:** C. L. Fonseka, A. G. T. A. Kariyawasam, S. D. A. L. Singhapura, C. M. de Silva, T. E. Kanakkahewa, I. G. T. M. Senarathna, C. K. Bodinayake

**Affiliations:** ^1^University Medical Unit, Teaching Hospital Karapitiya, Galle, Sri Lanka; ^2^Department of Internal Medicine, Faculty of Medicine, University of Ruhuna, Sri Lanka

In the article titled “Metastatic Renal Cell Carcinoma Presenting as Prolonged Pyrexia and Stauffer's Syndrome: Can a Routine Ultrasound Scan Fail to Detect a Renal Cell Carcinoma?” [1], the name of the third author was given incorrectly as S. A. G. L. Singhapura. The author's name should have been written as S. D. A. L. Singhapura. The revised authors' list is shown above.

The author's Contribution section should also be updated to “C. L. Fonseka, A. G. T. A. Kariyawasam, S. D. A. L. Singhapura, I. G. T. M. Senarathna, and C. K. Bodinayake investigated the case. C. M. de Silva, T. E. Kanakkahewa, and C. K. Bodinayake planned radiological investigation and prepared the manuscript. All authors were involved in editing the content and approved the final version for publication.”
